# Adolescents’ Perceptions of Household Chaos Predict Their Adult Mental Health: A Twin-Difference Longitudinal Cohort Study

**DOI:** 10.1177/09567976241242105

**Published:** 2024-05-08

**Authors:** Sophie von Stumm

**Affiliations:** Department of Education, University of York

**Keywords:** subjective household chaos, twin-difference design, adult mental health, family background inequality, adolescence, open materials, preregistered

## Abstract

This study tested whether adolescents who perceived less household chaos in their family’s home than their same-aged, same-sex sibling achieved more favorable developmental outcomes in young adulthood, independent of parent-reported household chaos and family-level confounding. Data came from 4,732 families from the Twins Early Development Study, a longitudinal, U.K.-population representative cohort study of families with twins born in 1994 through 1996 in England and Wales. Adolescents who reported experiencing greater household chaos than their sibling at the age of 16 years suffered significantly poorer mental-health outcomes at the age of 23 years, independent of family-level confounding. Mental-health predictions from perceived household chaos at earlier ages were not significant, and neither were predictions for other developmental outcomes in young adulthood, including socioeconomic status indicators, sexual risk taking, cannabis use, and conflict with the law. The findings suggest that altering children’s subjective perceptions of their rearing environments may help improve their adult mental health.

Household chaos refers to the level of disorganization or environmental confusion that children are exposed to in their family homes. Common characteristics of household chaos include a lack of family routines, the absence of predictability and structure in daily activities, and an overly fast pace of family life (Matheny et al., 1995; Wachs & Evans, 2010). Numerous studies have shown that children who grow up in chaotic households suffer adverse developmental outcomes, including impaired social-emotional functioning, lower educational achievement, and greater behavioral problems (e.g., Evans, 2006; Marsh et al., 2020; Martin et al., 2012). Yet no prior study has tested whether children’s exposure to household chaos has a direct, long-term influence on their developmental outcomes in adulthood.

Because children actively construct their experiences and respond differently to the environment, they vary substantially in development even if they grow up in the same family (Plomin & Bergeman, 1991; Rivenbark et al., 2019). For example, in the majority of families, siblings differ in the grades that they receive in school (i.e., within-family differences; Plomin & Daniels, 1987; Selzam et al., 2019), even though the major source of children’s differences in school performance is family background (i.e., between-family differences; Heckman, 2006; von Stumm et al., 2022). Within-family discrepancies in school grades translate, among other things, into 27% of sibling pairs being discordant in qualifying for university-level studies (Ayorech et al., 2017), a factor that predicts a plethora of developmental outcomes throughout the life span (Mackenbach et al., 2008; Mäki et al., 2013).

Children’s and adolescents’ subjective perceptions predict their long-term well-being (Greven et al., 2009; Quon & McGrath, 2014), sometimes even more strongly so than their objective experiences (Danese & Widom, 2020). Yet associations between subjective experiences of the family home (i.e., within-family differences) and later developmental outcomes are often confounded by unmeasured variation in environmental and genetic factors that occur at the family level and are shared between siblings of the same family (i.e., between-family differences that drive family-level confounding). By testing whether twins’ differences in their subjective experiences of household chaos within families predict their developmental differences in adulthood, the twin-difference design controls for all variation that occurs between families and that typically compromises the interpretation of correlational findings (Rivenbark et al., 2020). Previously, a twin-difference study showed, for example, that 18-year-old adolescents who viewed their families as having higher social status than their co-twins were concurrently less likely to be convicted of a crime; not in education, employment, or training (NEET); and had fewer mental-health problems (Rivenbark et al., 2020). Although the cross-sectional nature of this earlier study does not allow ruling out reverse causation (e.g., adolescents who have mental-health problems may rate their families as having lower social standing), its findings suggested that altering adolescents’ subjective social status may lead to greater well-being and social mobility (Rivenbark et al., 2020).

Improving the life chances of children from disadvantaged family homes is difficult because these children are exposed to numerous psychosocial and physical environmental risks that cumulate in their effects on development (Evans, 2004; Heckman, 2006; von Stumm, 2022). Altering children’s perceptions of household chaos is potentially a feasible and effective way for disrupting the transmission of family background inequality (Evans, 2006). Finding that children’s subjective experiences of household chaos predict their developmental trajectories independently of family-level confounding would advocate investing in trials for yet-to-be developed interventions that target children’s chaos-related perceptions, akin to interventions that teach children that their intellectual abilities can be developed to improve their school outcomes (e.g., Yeager et al., 2019). By contrast, if associations between subjective experiences of household chaos and developmental outcomes can be fully attributed to genetic and environmental factors that siblings share within their family, then altering children’s perceptions of chaos is unlikely to produce developmental changes.

The long-term effects of children’s subjective and their parents’ reported household chaos on adult development have not been previously tested, although the negative influence of household chaos on child and adolescent development has been broadly demonstrated (for a review, see Marsh et al., 2020). This study capitalized on twins’ individual, subjective reports of household chaos at the ages of 9, 12, 14, and 16 years (i.e., different report by each twin in the family or within-family difference); their parents’ ratings of household chaos at the ages of 9, 12, and 14 years (i.e., same report for both twins or between-family difference); and measures of twins’ developmental outcomes at the age of 23 years. We tested whether adolescents (a) who perceived their family homes as more chaotic and (b) whose parents reported greater household chaos had worse developmental outcomes in young adulthood. We then assessed whether the prediction from subjective household chaos remained significant after controlling for relevant confounders, including previous, parent-reported household chaos and family socioeconomic status (SES), as well as after leveraging the twin-difference design that controls for all family-level confounding resulting from environmental and genetic factors that are shared by two siblings in a family. We predicted that subjective experiences of household chaos would continue to significantly predict adulthood development, even after controlling for family-level confounding. Previous studies have suggested that subjective perceptions of family background characteristics manifest in adolescence as meaningful predictors of education, mental health, and well-being (Rivenbark et al., 2020; Steinberg & Morris, 2001), whereas the effects of family-level household chaos (i.e., “objective” or parent-reported) are thought to exert a stable influence on development from early childhood onward (Marsh et al., 2020). We therefore tested whether at what ages predictions from child- compared with parent-reported household chaos became salient for young adulthood outcomes.

**Statement of Relevance**

Disrupting the link between inequalities in children’s family home experiences and their long-term developmental outcomes is the goal of researchers, practitioners, and policymakers across the globe. Alas, effective interventions remain scarce because associations between family home experiences and developmental outcomes are confounded by unmeasured environmental and genetic factors that are shared between siblings within the same family. The twin-difference design offers a stringent test of whether associations between subjective family home experiences and developmental outcomes are likely causal or due to family-level confounding. Here, evidence for a causal pathway was found: Adolescents who reported experiencing greater household chaos than their same-age, same-sex sibling, who grew up in the same family home, suffered poorer mental-health outcomes in young adulthood. If confirmed by future research, efforts to improve mental health in young adulthood should prioritize the development of interventions that alter subjective perceptions of children’s family home experiences.

## Open Practices Statement

This study’s methods, analysis plan, and hypotheses were preregistered prior to analysis on the OSF at https://osf.io/knyvm. All questionnaires and web tests used in this study are publicly available from the Twins Early Development Study (TEDS) data dictionary at https://www.teds.ac.uk/datadictionary/home.htm. Data for this study can be accessed at https://www.teds.ac.uk/researchers/teds-data-access-policy. The analysis code for this study is not publicly accessible.

## Method

### Sample

Data came from the TEDS, which recruited families with twins born between 1994 and 1996 in England and Wales (Rimfeld et al., 2019). At the twins’ age of 18 months, 13,759 families participated; during this first assessment, data were collected on demographics, pregnancy, childbirth, and zygosity. Zygosity was assigned using a parent-reported questionnaire of physical similarity, which is more than 95% accurate compared with DNA testing (Price et al., 2000). For cases in which zygosity was unclear, DNA testing was conducted.

At the twins’ age of 9 years, approximately 3,400 twin pairs contributed data, at 12 years approximately 5,900 pairs, at 14 years approximately 3,000 pairs, at 16 years approximately 6,000 pairs, and at 23 years approximately 3,300 pairs. The number of twin pairs contributing data varied across assessment waves because of funding restrictions, with approximately half of the families being invited to participate at the twins’ ages of 9 and 14 years than at the ages of 12 and 16 years. Twins who suffered from severe medical problems currently or at birth (e.g., postnatal surgery) and whose mothers reported severe medical problems during pregnancy were excluded from the analyses. The analysis sample included all same-sex twin pairs who reported on household chaos at least once, resulting in 4,732 complete twin pairs and 9,477 individual twins. We excluded opposite-sex twin pairs to rule out that our findings on subjective household chaos were confounded by gender differences within twin pairs.

TEDS families are broadly representative of other U.K. families in the 1990s (Rimfeld et al., 2019). For example, 93% of the TEDS families identified as White versus 93% of the U.K. families, and 44% of the TEDS mothers were employed at twins’ ages 2 through 4 years versus 50% of the U.K. mothers in the 1990s (Rimfeld et al., 2019). Using *z*-scores (*M* = 0, *SD* = 1), the analysis sample’s average SES, a composite of mothers’ and fathers’ education and occupation, as well as mothers’ age at first birth (not necessarily the twins; see details below), the mean was 0.12 (*SD* = 0.99; *N* = 4,436 families), which is minimally higher than the overall sample’s SES (*M* = 0, *SD* = 1), suggesting that the analysis sample was representative in SES of the overall sample.

This study met the ethical guidelines and legal requirements of the University of York. Parents and twins provided written informed consent prior to data collection. TEDS project approval (05.Q0706/228) was granted by the ethics committee for the Institute of Psychiatry, Psychology and Neuroscience at King’s College London.

### Measures

#### SES

Family SES was assessed by measures at first contact when the TEDS twins were 18 months and when they were 7, 9, and 16 years old. At first contact (SES Index 1), age 7 (SES Index 2), and age 16 (SES Index 4), parents reported their highest educational qualifications and occupation. Educational qualifications were assessed on an 8-point scale from 1 (*no formal education*) to 8 (*postgraduate qualifications*). Occupation was inferred on the basis of a standard classification (Office of Population and Census Surveys, 1991) that used reports of employment status, job title, employment type, and whether parents needed special qualifications for their role. At 9 years (SES Index 3) and 16 years, family income was assessed; parents reported their annual household income before tax on an 11-point scale from 1 (*under £4,500*) to 11 (*more than £100,000*). For SES Indexes 1 through 3, standardized mean scores were calculated and averaged at each assessment age. Previous analyses of these data showed that correlations (*r*) between these estimates were .77 for SES Index 1 and 2, .55 for SES Indexes 1 and 3, and .57 for SES Indexes 2 and 3 (Hanscombe et al., 2012). Correlations with SES Index 4 have not been previously reported and were .70 with SES Index 1, .76 with SES Index 2, and .65 with SES Index 6 in the current analysis sample. We summed the four indexes to achieve one SES composite score for each participant.

#### Household chaos

At 9, 12, 14, and 16 years, the twins rated household chaos using a short version of the Confusion, Hubbub, and Order Scale (CHAOS), and at the twins’ ages of 9, 12, and 14 years their parents (i.e., mother or father per twin pair) also completed the short version of the CHAOS (Matheny et al., 1995). The full CHAOS has high internal consistency (Cronbach’s α = .79) and test–retest reliability (*r* ≥ .70) across a 12-month period (Dumas et al., 2005; Matheny et al., 1995). The CHAOS was administered as part of a larger battery of measures in a booklet mailed to each of the twins and their parents. The short form of the CHAOS assesses the level of routine, noise, and general environmental confusion with six items: “I have a regular bedtime routine” (reverse coded), “You can’t hear yourself think in our home,” “It’s a real zoo in our home,” “We are usually able to stay on top of things” (reverse coded), “There is usually a television turned on somewhere in our home,” and “The atmosphere in our house is calm” (reverse coded). The children and parents rated the extent to which they agree as *not true, quite true*, or *very true*. Cronbach’s alphas ranged from .56 to .60 for children’s ratings of household chaos across ages 9 through 16 years (cf. Hannigan et al., 2017), and Cronbach’s alphas for parents’ ratings ranged from .57 to .65 across ages 9 through 14 years in the analysis sample.

#### Young adulthood outcomes

At age 23, participants completed web-based questionnaires and reported a broad range of outcomes, including educational attainment, occupational status, earnings, substance use, sexual risk taking, conflict with the law, and mental health.

#### Educational attainment

Twins reported their highest educational qualification, which was recoded into ordinal variables on an 11-point scale from *no qualifications* (1) to *doctoral degree* (11). If twins were still in education, they reported the highest qualification they were working toward. We inferred the highest educational level from the highest response reported for either completed or working toward.

#### Employment status

Twins reported whether they were currently studying, working, doing an apprenticeship or other employment training, taking a gap year or traveling, being unemployed, or a full-time parent. To create a binary measure, those who reported being unemployed were coded as 0, reflecting NEET status; and those working, studying, and doing apprenticeship or employment training were coded as 1, reflecting being employed, in school, or in training. Missing data values were assigned to those on a gap year or identifying as full-time parents.

#### Benefit status

Participants reported whether they received any benefits, including housing benefits, child benefits, child tax credit, working tax credit, jobseekers’ allowance, income support, and employment and support allowance. We coded the responses as 1 for people who indicated that they received at least one type of benefit and 0 for those who did not receive any benefits.

#### Income

Twins who were currently working reported how much money they earned after taxes in an average month on a 9-point scale with £500 increments from *£0–500* (1) to *more than £4,000* (9).

#### Anxiety

Twins completed 10 items from the Adult Severity Measure for Generalized Anxiety Disorder (Craske et al., 2013), indicating how often they had experienced each situation during the past 7 days on a 5-point scale from *never* (0) to *all of the time* (4). An example item reads “I have had thoughts of bad things happening, such as family tragedy, ill health, loss of a job or accidents.” Scores were summed.

#### Alcohol use

Twins who indicated they had had a whole drink completed 10 items adapted from the Alcohol Use Disorders Identification Test developed by the World Health Organization. An example question reads: “During the past year, how often have you had six or more units of alcohol on one occasion?” Twins responded on a 5-point scale from *never/almost never* (0) to *daily/almost daily* (4). Scores were summed. Twins who indicated they had not had a whole drink before received a score of 0.

#### Cannabis use

Twins who indicated they had tried cannabis answered this question: “In the last 12 months how often have you used cannabis?” Twins responded on a 6-point scale from *not in the last 12 months* (0) to *daily or almost daily* (5).

#### Sexual risk taking

Twins who indicated they had had sexual intercourse completed four items designed by TEDS researchers to assess (non)safe sex practices. Twins reported the age when they first had sexual intercourse on a 6-point scale from *12 or younger* (5) to *17 or older* (0). Twins also reported how many sex partners they have had on a 5-point scale from *1 person* (1) to *15 or more* (5) and how often they used a condom or other contraception on a 5-point scale from *never* (0) to *always* (4). Finally, they reported how often they had been diagnosed with a sexually transmitted disease on a 3-point scale from *never* (0) to *2 or more times* (2). Scores were summed, with higher scores indicating higher sexual risk taking. Those who reported not to have had intercourse received a sexual risk score of 0.

#### Aggression

Physical and verbal aggression were assessed with the eight items from the Brief Aggression Questionnaire using a 5-point scale from *strongly disagree* (1) to *strongly agree* (5; Webster et al., 2014). An example item reads “Given enough provocation, I may hit another person.” Scores were summed and averaged across items.

#### Antisocial behavior

Twins indicated whether and how often they engaged in 11 antisocial behaviors on a 5-point scale from *no* (0) to *more than 10 times* (4). The statements were adapted from the Edinburgh Study of Youth Transitions and Crime (McAra & McVie, 2010) and included destroying property and selling illegal drugs. An overall score was computed by summing responses.

#### Conflict with the law

Twins completed four questions designed by TEDS researchers, answering *yes* (1) or *no* (0) to indicate whether they had been cautioned by the police, whether they had been arrested, and whether they had ever been sentenced to prison, as well as reporting how many times they had been arrested (1 = *once*, 2 = *2–4 times*, 3 = *5 or more times*). Scores were summed.

#### Depression

Twins completed the short version of the Mood and Feelings Questionnaire (Angold et al., 1995), an eight-item screening tool for depression that assesses feelings and behaviors that characterize low well-being. Participants indicated how much each statement applied to their experiences over the last 2 weeks on a 3-point scale from 0 (*not true*) to 2 (*very true*). An example item reads “I didn’t enjoy anything at all.” Scores were summed, with higher values indicating greater depression.

#### Self-control

Twins completed the Brief Self-Control Survey (Tangney et al., 2004), a six-item scale that assesses how well respondents can override distractions using a 5-point scale from *not at all* (0) to *very much* (4). An example item reads “I am good at resisting temptation” and “I have a hard time breaking bad habits” (reverse coded). Scores were summed.

### Statistical analysis

All analyses were carried out in R Version 4.2.1 (R Core Team, 2022). To deal with missing data in our linear regression models and co-twin designs, full information maximum likelihood was applied using lavaan Version 0.6-12 (Rosseel, 2012).

We focused our initial analyses on adolescents’ perceptions of household chaos that they reported at the age of 16 years for two reasons. First, subjective perceptions of family background characteristics are thought to be salient predictors of adult development from adolescence (Rivenbark et al., 2020; Steinberg & Morris, 2001), but it is yet unknown whether they are also predictive earlier during childhood. Second, adolescents’ reports of household chaos at the age of 16 years were closest in time to their reports of developmental outcomes at the age of 23 years. If subjective perceptions of household chaos predicted young adulthood outcomes, these relations would be most likely to be evident for assessments that were closest in time. To test whether adolescents who perceived their family homes as more chaotic had less favorable developmental outcomes in adulthood, we fitted a series of linear regression models for each adulthood outcome, including measures of SES attainment, sexual risk taking, mental health, substance use, and conflict with the law. For all models, regression error terms were clustered at the family level to account for the correlation of error terms within families. All outcomes in adulthood were continuous except for two binary ones (i.e., employment and benefit status), which can be modeled in lavaan (Rosseel, 2012).

Model 1 included twins’ subjective household chaos at age 16 as a predictor of an adulthood outcome at age 23. Model 2 added three covariates that are likely to confound associations between subjective household chaos and adult development, specifically family SES, twins’ gender, and parent-reported household chaos at twins’ age of 14 years (parents did not report household chaos at twins’ age of 16 years). Model 3 used the twin-difference design, in which relative (signed) within-twin-pair differences in the adulthood outcome were predicted from their within-twin-pair differences in subjective household chaos. In additional analyses, we compared our twin-difference models in the overall sample to those in subsamples of monozygotic and dizygotic twins, respectively. We also conducted multigroup models that restricted the regression paths from twins’ differences in the perception of household chaos at age 16 to their differences in adulthood outcomes to be equal across zygosity. The model results did not differ significantly across the monozygotic, dizygotic, and overall sample of twins; we therefore report the results based on the overall sample herein and the results of the additional analyses in the Supplemental Material available online (see Tables S8 and S9).

To test when the prediction from subjective household chaos for adulthood outcomes became salient during childhood, we fitted the models described above (Models 1–3) for twins’ ratings of chaos at the ages of 9, 12, and 14 years and compared the results to those derived using twins’ chaos ratings at the age of 16. For Model 2, we added concurrently assessed parent-reported household chaos as a covariate (i.e., when prediction from child-reported household chaos at age 12 was tested, the parent-reported household chaos at the same age was added as covariate, not at age 14, which was used in the models above). To test when the prediction from family-level household chaos for adulthood outcomes became salient, we fitted linear regression models with parent-reported household chaos as a predictor at the ages of 9, 12, and 14 years.

Finally, we explored whether the predictions from child- and parent-reported household chaos had additive effects across ages 9 through 16 years. To this end, we summed twins’ reports of household chaos across assessment ages, as well as their differences in subjective household chaos, and we also summed parent-reported household chaos across assessment ages. We then fitted Models 1 through 3 as described above for each adulthood developmental outcome.

## Results

Children’s subjective household chaos correlated (r) between .35 and .54 from ages 9 through 16 years, confirming both stability and change in their perception of household chaos over time (Table S1). Parents’ ratings correlated between .58 and .64 across assessment times, indicating that parents’ perceptions of household chaos were more stable than their children’s. Children’s and parents’ chaos ratings differed from each other, with correlations ranging from .34 to .55. Between two twins of a pair, household chaos ratings correlated between .29 and .61 across assessments (Table S2), confirming that there was within-family variation in children’s subjective household chaos.

### Do adolescents’ subjective perceptions of chaos at age 16 predict developmental outcomes in young adulthood at age 23?

Table 1 shows the prediction of developmental outcomes in young adulthood, assessed at the age of 23 years, from subjective household chaos, which was reported by adolescents when they were 16 years old, across unadjusted models, models adjusted for confounders (i.e., gender, family SES, and parent-rated chaos when the twins were 14 years old), and twin-difference models (i.e., adjusted for all family-level confounding). Adolescents who reported experiencing greater household chaos had on average worse developmental outcomes in young adulthood across domains. In the unadjusted models, this effect was significant for 11 of 13 outcomes, with effect sizes (*R*^2^) ranging from .009 for income to .051 for self-control. After adjusting for confounders, these 11 associations remained significant, suggesting robust effects of adolescents’ subjective experiences of household chaos on their later adulthood development, beyond the objective conditions they grew up in.

**Table 1.** Prediction of Developmental Outcomes in Young Adulthood From Adolescents’ Subjective Household Chaos at the Age of 16 Years

	Model 1: unadjusted			Model 2: with covariates			Model 3: twin difference		
	β	95% CI	*R* ^2^	β	95% CI	*R* ^2^	β	95% CI	*R* ^2^
Education	−**.198**	**[−.241, −.156]**	.039	**−.076**	**[−.127, −.025]**	.190	.036	[−.044, .116]	.001
Employment status	−.037	[−.079, .005]	.001	−.045	[−.092, .003]	.005	−.043	[−.108, .022]	.002
Income	**−.092**	**[−.143, −.042]**	.009	**−.076**	**[−.132 −.020]**	.037	−.030	[−.122, .062]	.001
Benefits	**.111**	**[.064, .158]**	.012	**.082**	**[.030, .133]**	.033	.008	[−.063, .076]	.000
Depression	**.211**	**[.169, .253]**	.045	**.181**	**[.133, .230]**	.064	**.106**	**[.041, .170]**	.011
Self-control	**−.226**	**[−.267, −.185]**	.051	**−.243**	**[−.291, −.195]**	.067	**−.140**	**[−.206, −.073]**	.019
Anxiety	**.181**	**[.134, .228]**	.033	**.162**	**[.108, .216]**	.056	**.124**	**[.050, .198]**	.015
Antisocial behavior	**.135**	**[.086, .184]**	.018	**.123**	**[.063, .183]**	.035	**.129**	**[.062, .197]**	.017
Aggression	**.206**	**[.164, .247]**	.042	**.176**	**[.128, .223]**	.076	**.140**	**[.071, .209]**	.020
Alcohol use	**.117**	**[.066, .168]**	.014	**.129**	**[.070, .189]**	.039	**.129**	**[.055, .203]**	.017
Cannabis use	.038	[−.031, .106]	.001	.032	[−.042, .107]	.023	.094	[−.028, .216]	.009
Sexual risk taking	**.132**	**[.084, .179]**	.017	**.082**	**[.027, .137]**	.026	.017	[−.065, .099]	.000
Conflict with law	**.130**	**[.081, .180]**	.017	**.090**	**[.027, .152]**	.037	.060	[−.021, .140]	.004

Note: Associations with 95% CIs excluding 0 are shown in bold. Employment status and benefits were binary variables; all others were continuous. The covariates in Model 2 are gender, family socioeconomic status, and parent-rated chaos when the twins were 14 years old. Regression error terms were clustered at the family level in all models. The number of used observations (*N*) across models and outcomes ranged from 4,068 to 9,477. CI = confidence interval.

In the twin-difference models, which have greater purchase on causality inferences than simple confounder-adjusted models because they control for all genetic and environmental factors that two siblings in a family share, the prediction from adolescents’ subjective household chaos experiences remained significant for six outcomes. All six pertained to mental health, including depression, self-control (negative), anxiety, antisocial behavior, aggression, and alcohol use. Effect sizes (*R*^2^) ranged from .011 for depression to .020 for aggression, suggesting that the household chaos that children experience independent of any family-level confounding accounted for 1% to 2% of the variance in mental-health outcomes in young adulthood.

### When during childhood does the prediction from subjective household chaos for adult developmental outcomes become salient?

**Fig. 1.**
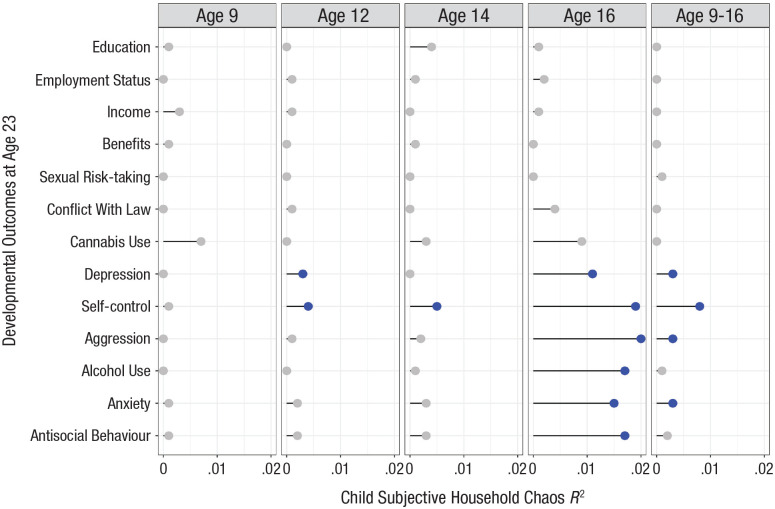
Prediction of developmental outcomes at the age of 23 years from subjective perceptions of household chaos at ages 9 through 16 years. *R*^2^ values are taken from twin-difference models. Blue points index *R*^2^ predictions that were significant (*p* < .050); gray points reflect nonsignificant predictions.

At age 14, adolescents’ ratings of household chaos significantly predicted 11 of 13 developmental outcomes in young adulthood, and eight of these associations remained significant after adjusting for covariates (i.e., gender, family SES, and parent-reported household chaos at twins’ age 14 years; *N* = 4,985–9,477; Table S3). Using the twin-difference design, one prediction—for self-control—was associated with 95% confidence intervals (CIs) that excluded 0 (Fig. 1). The corresponding effect size was a quarter that observed for the prediction from subjective household chaos at the age of 16 years (*R*^2^ = .005 vs .019).

At age 12, child-reported household chaos significantly predicted 11 of 13 outcomes in adulthood, and six of these associations remained significant after adjusting for covariates (i.e., gender, family SES, and parent-reported household chaos at twins’ age of 12 years; *N* = 8,000–9,477; Table S4). In the twin-difference model, two predictions—for depression and self-control—were associated with 95% CIs that excluded 0. Corresponding effect sizes (*R*^2^ values) were small, accounting for 0.3% and 0.4% of the variance in depression and self-control, respectively, independent of any family-level confounding (Fig. 1).

At age 9, children’s ratings of household chaos significantly predicted 10 of the 13 developmental outcomes in adulthood, and three of these associations remained significant after adjusting for confounders (i.e., gender, family SES, and parent-reported household chaos at twins’ age of 9 years; *N* = 5,098–9,477; Table S5). However, no prediction was associated with a 95% CI excluding 0 in the twin-difference models (Fig. 1).

### When during childhood do associations between parent-reported household chaos and adult developmental outcomes become salient?

**Fig. 2.**
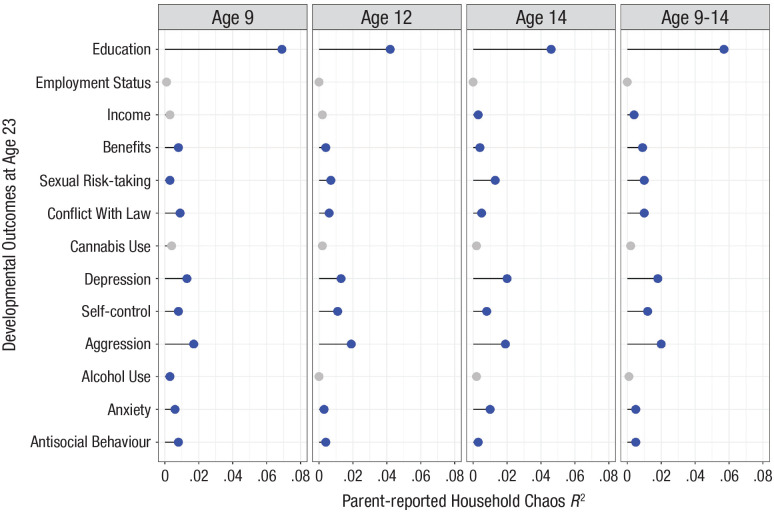
Prediction of developmental outcomes at the age of 23 years from parent-reported household chaos at children’s ages 9 through 14 years. *R*^2^ values are from models that included only parent-reported household chaos as predictors, which is the same for two siblings in a family (i.e., between-family differences). Blue points index *R*^2^ predictions that were significant (*p* < .050); gray points reflect nonsignificant predictions.

In contrast to predictions from children’s subjective household chaos, predictions from parent-reported chaos, which was the same for two siblings in a family, were salient already at age 9, with consistent effect sizes across assessment ages (Fig. 2). For example, parent-reported chaos at children’s age 9 years explained 7% of the variance in educational attainment, 4% at children’s age 12 years, and 5% at adolescents’ age 14 years. Effect sizes for predictions from parent-reported household chaos were significant for 10 of 13 adult developmental outcomes at age 9, and these associations were also observed at the ages of 12 and 14 years with two exceptions. The prediction of income became significant only at the age of 14 years, whereas the prediction of alcohol use was significant only at the age of 9 years but not thereafter. Effect sizes were largest for predicting educational attainment but smaller for other adult developmental outcomes (Fig. 2). For the six mental-health outcomes, the prediction effect sizes (*R*^2^) from parent-reported household chaos averaged .010 across assessment ages, accounting for 1% of the variance. By comparison, the average prediction for adult mental health from children’s subjective household chaos at age 16, independent of all family-level confounding, accounted for 1.65% of the variance (Fig. 1).

**Fig. 3.**
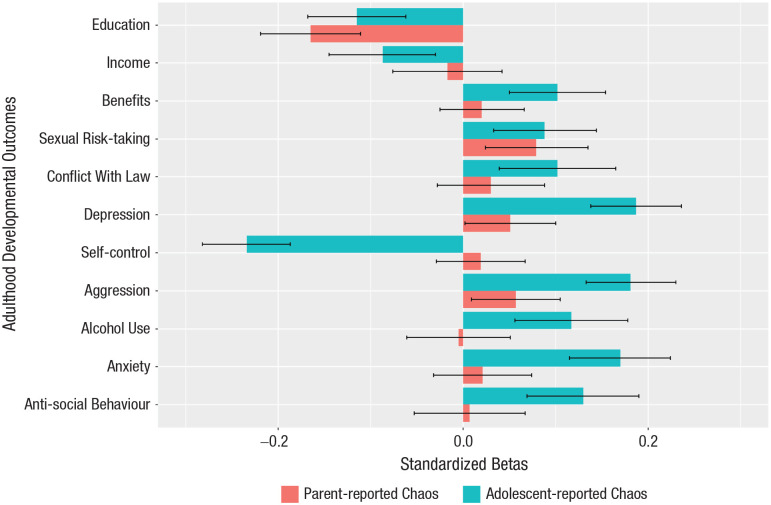
Adolescent- and parent-reported household chaos predict developmental outcomes in young adulthood. Bars represent standardized regression coefficient estimates (blue for child-reported chaos and red for parent-reported chaos). Models included only adolescent- and parent-reported household chaos at the age of 16 and 14 years, respectively, as predictors. Models were not significant for cannabis use and employment status; these outcomes were therefore omitted from the graph. Error bars indicate 95% confidence intervals. Regression error terms were clustered at the family level to account for correlation of the within-family error terms. The number of used observations (*N*) varied by the availability of data on the outcome variable from 5,318 to 6,404.

Figure 3 directly compares the predictions from adolescent- and parent-reported household chaos for adulthood outcomes at age 23. The prediction from adolescent-reported household chaos at age 16 was significantly greater (*p* < .050) than that from parent reports for the six mental-health outcomes, including depression, self-control, aggression, anxiety, alcohol use, and antisocial behavior, with adolescents who reported experiencing greater household chaos suffering worse mental health in young adulthood. We observed no significant differences between adolescent- and parent-reported household chaos for the other developmental outcomes.

### Are children’s subjective experiences of household chaos additive across ages in their prediction of adult outcomes?

The stability of twins’ differences in their subjective experience of household chaos increased over time, ranging from *r* = .06 between the ages of 9 and 12 years to *r* = .25 between the ages of 14 and 16 years. Children’s cumulative subjective household chaos (i.e., sum scores across ages 9, 12, 14, and 16 years) significantly predicted 12 of 13 developmental outcomes in young adulthood, and nine of these associations remained significant after adjusting for covariates (i.e., gender and family SES as before and cumulative parent-reported household chaos across ages 9, 12, and 14 years; *N* = 9,374–9,4877). In the twin-difference models, four predictions—for depression, self-control, anxiety, and aggression—were associated with 95% CIs that excluded 0 (Table S6; Fig. 1). Effect sizes of these predictions (*R*^2^) ranged from .003 to .008 averaged 0.4% of the variance in the mental-health outcomes, less than a quarter of the average prediction from subjective chaos at age 16 had been (Table 1).

### Is parent-reported household chaos additive across ages in its prediction of adult outcomes?

Parent-reported, cumulative subjective household chaos (i.e., sum scores across the ages of 9, 12, and 14 years) significantly predicted 10 of 13 developmental outcomes in young adulthood (Fig. 2; *N* = 9,109–9,240; Table S7). Effect sizes (*R*^2^) of the cumulative predictions ranged from 0 to .057, accounting on average for 1.17% of the variance across adult developmental outcomes.

## Discussion

Household chaos—characterized by crowding, noise, unpredictability, and a lack of routines—describes the physical and social environment that some children are exposed to in their family homes (e.g., Evans, 2006; Marsh et al., 2020; Wachs & Evans, 2010). We showed here for the first time that adolescents who reported experiencing more household chaos than their same-sex, same-age sibling, who grew up with them at the same time in the same family home, suffered worse mental-health outcomes in young adulthood (Fig. 1). We also found that these within-family differences in adolescents’ subjective experiences of household chaos were stronger predictors of adult mental health than parents’ reports of household chaos, which capture between-family differences and are the same for two siblings of a family. Finally, we observed that although parents’ reports of household chaos predicted developmental outcomes other than adult mental health, these predictions were not significantly different from those from adolescents’ subjective perceptions of household chaos. Together, our findings suggest that subjective experiences of the family home environment, more than the objective realities of the household chaos that children are reared in, drive their mental-health differences in young adulthood (cf. Danese & Widom, 2020).

Predictions from adolescents’ subjective household chaos for later mental health accounted for 1% to 2% of the variance in depression, self-control, anxiety, aggression, antisocial behavior, and alcohol use at the age of 23 years. Although these effect sizes may seem small, they are likely to be robust because they were reliably estimated independent of family-level confounding and were consistent across all six measures of adult mental health that were available in this study (Funder & Ozer, 2019; Götz et al., 2022).

The link between adolescents’ subjective experiences of household chaos and adult mental health was evident at age 16 but weaker at younger ages. By contrast, parents’ reports of family-level household chaos were consistently associated with a broad range of adult developmental outcomes from children’s ages 9 through 14 years, a finding that aligns with previous research (Evans, 2006; Marsh et al., 2020; Wachs & Evans, 2010). We observed neither for child- nor parent-reported household chaos evidence that effects on adult mental-health outcomes accumulated across ages. Future research is needed to identify the ages when intervention efforts to change perceptions of household chaos will be most impactful because these may be different from the age at which associations between perceptions and mental health become salient.

Predictions from children’s subjective household chaos before the age of 16 years were only repeatedly significant for self-control in young adulthood, independent of family-level confounding. Prior studies have shown that self-control is a particularly powerful predictor of adult health, wealth, and crime and that the association between self-control and a wide range of adult developmental outcomes is already evident in early childhood (Moffitt et al., 2011). Thus, disentangling the developmental interplay between children’s subjective perceptions of household chaos and their self-control should be a priority for future research that seeks to identify ways for improving adult developmental outcomes.

### Limitations

The current study has several strengths, including a longitudinal twin study design; multi-informant, repeated measures of household chaos; and the assessment of a broad range of adult developmental outcomes. It is also not without weaknesses. First, the twin-difference design does not exclude the possibility of unshared confounding factors that affect both adolescents’ perceptions of their family home environments and their adulthood outcomes, for example, when emerging mental-health problems influence the experience of household chaos. The current study’s focus was predicting adult developmental outcomes from adolescents’ subjective household chaos, and further studies are needed to elucidate whether, why, and by which factors associations between subjective perceptions of household chaos and adult mental health may be confounded. Future research is also needed to explore the generalizability of the current findings for adolescents’ and children’s perceptions of environments other than household chaos. Second, household chaos was reported on the six-item short form of the original 15-item CHAOS measure (Matheny et al., 1995). Because it is unclear to what extent the short CHAOS form captures the construct space of the longer version, findings that rely on the short CHAOS form must be interpreted with caution (Larsen et al., 2022), even though the short CHAOS form is frequently used in research (Hannigan et al., 2017; Hanscombe et al., 2011; Hart et al., 2007; Seidler & Ritchie, 2018). Third, all adulthood outcomes in the current study were self-reported, but clinical diagnoses or physician-reported mental-health outcomes were not available. Finally, the twin cohort study whose data were analysed here, the TEDS, has suffered some attrition, as is typical in longitudinal studies, with 92% of the twin families who contributed when the twins were 7 years old also contributing when the twins were 23 years old (Rimfeld et al., 2019). Sample characteristics varied negligibly over time (Rimfeld et al., 2019), suggesting minimal biases because of attrition.

## Conclusions

Improving the life chances of children who experience adverse family home environments is key to disrupting the intergenerational perpetuation of background inequality in life-span development. However, identifying the factors that drive the link between children’s early family home experiences and their later developmental outcomes is difficult because of the threat of family-level confounding. Using twin-difference models that control for family-level confounding, we showed here that adolescents’ subjective experiences of the household chaos in their family homes significantly predicted their adult mental health. This result suggests that altering children’s subjective perceptions of their family’s household chaos is potentially an effective way for improving their mental-health outcomes in adulthood.

## Supplemental Material

sj-docx-1-pss-10.1177_09567976241242105 – Supplemental material for Adolescents’ Perceptions of Household Chaos Predict Their Adult Mental Health: A Twin-Difference Longitudinal Cohort Study Supplemental material, sj-docx-1-pss-10.1177_09567976241242105 for Adolescents’ Perceptions of Household Chaos Predict Their Adult Mental Health: A Twin-Difference Longitudinal Cohort Study by Sophie von Stumm in Psychological Science

## Transparency

*Action Editor:* Daniela Schiller

*Editor:* Patricia J. Bauer

*Author Contributions*

**Sophie von Stumm:** Conceptualization; Formal analysis; Funding acquisition; Investigation; Methodology; Project administration; Software; Visualization; Writing – original draft; Writing – review & editing.

**Open Practices:** This article has received the badges for Open Materials and Preregistration. More information about the Open Practices badges can be found at http://www.psychologicalscience.org/publications/badges.
