# Supplementary material for: Adolescents’ Perceptions of Household Chaos Predict Their Adult Mental Health: A Twin-Difference Longitudinal Cohort Study
Source: Psychol Sci. 2024 May 8;35(7):736–48. doi: 10.1177/09567976241242105 (PMC13020938; doi:10.1177/09567976241242105)
Supplement: sj-docx-1-pss-10.1177_09567976241242105 – Supplemental material for Adolescents’ Perceptions of Household Chaos Predict Their Adult Mental Health: A Twin-Difference Longitudinal Cohort Study [file sj-docx-1-pss-10.1177_09567976241242105.docx]

**Supplementary Materials**

Adolescents’ perceptions of household chaos predict their adult mental health: A twin difference longitudinal cohort study

Table of Contents

[Table S1. Correlations between twins’ and parents’ household chaos ratings across ages 9 through 16 years 2](#_Toc156643367)

[Table S2. Within-twin pair correlations of household chaos ratings 3](#_Toc156643368)

[Table S3. Prediction of adulthood developmental outcomes at age 23 years from child-reported, subjective household chaos at age 14 years 4](#_Toc156643369)

[Table S4. Prediction of adulthood developmental outcomes at age 23 years from child-reported, subjective household chaos at age 12 years 6](#_Toc156643370)

[Table S5. Prediction of adulthood developmental outcomes at age 23 years from child-reported, subjective household chaos at age 9 years 8](#_Toc156643371)

[Table S6. Prediction of adult developmental outcomes from children’s subjective household chaos from age 9 through 16 years 10](#_Toc156643372)

[Table S7. Prediction of adult developmental outcomes from parent-reported household chaos at children’s ages 9 through 14 years 11](#_Toc156643373)

[Additional analyses 11](#_Toc156643374)

[Table S8. Twin difference model predictions of twins’ differences in subjective household chaos at age 16 for adult outcomes in monozygotic versus dizygotic twins 12](#_Toc156643375)

[Table S9: Multi-group model fit comparisons across monozygotic and dizygotic twins 13](#_Toc156643376)

# Table S1. Correlations between twins’ and parents’ household chaos ratings across ages 9 through 16 years

|  |  |  | 1 | 2 | 3 | 4 | 5 | 6 | 7 |
| --- | --- | --- | --- | --- | --- | --- | --- | --- | --- |
| 1 | Twins | Chaos age 9 | - |  |  |  |  |  |  |
| 2 |  | Chaos age 12 | .43 | - |  |  |  |  |  |
| 3 |  | Chaos age 14 | .37 | .51 | - |  |  |  |  |
| 4 |  | Chaos age 16 | .35 | .42 | .54 | - |  |  |  |
| 5 | Parents | Chaos age 9 | .53 | .43 | .39 | .34 | - |  |  |
| 6 |  | Chaos age 12 | .40 | .55 | .42 | .38 | .63 | - |  |
| 7 |  | Chaos age 14 | .38 | .42 | .49 | .42 | .58 | .64 | - |

# Table S2. Within-twin pair correlations of household chaos ratings

|  | Chaos age 9 T2 | Chaos age 12 T2 | Chaos age 14 T2 | Chaos age 16 T2 |
| --- | --- | --- | --- | --- |
| Chaos age 9 T1 | .60 | .40 | .35 | .29 |
| Chaos age 12 T1 | .41 | .61 | .41 | .37 |
| Chaos age 14 T1 | .38 | .40 | .54 | .42 |
| Chaos age 16 T1 | .32 | .35 | .45 | .53 |

Note. T1 is twin 1, and T2 is twin 2.

# Table S3. Prediction of adulthood developmental outcomes at age 23 years from child-reported, subjective household chaos at age 14 years in regression models

|  | Model | β | S.E. | p | CI 95% | | R2 |
| --- | --- | --- | --- | --- | --- | --- | --- |
| Education | Model 1 | -0.173 | 0.021 | 0.000 | -0.215 | -0.131 | 0.03 |
|  | Model 2 | -0.067 | 0.023 | 0.004 | -0.112 | -0.021 | 0.188 |
|  | Model 3 | 0.061 | 0.032 | 0.059 | -0.002 | 0.125 | 0.004 |
| Depression | Model 1 | 0.181 | 0.020 | 0.000 | 0.142 | 0.220 | 0.033 |
|  | Model 2 | 0.150 | 0.023 | 0.000 | 0.105 | 0.195 | 0.057 |
|  | Model 3 | 0.012 | 0.028 | 0.664 | -0.043 | 0.067 | 0. |
| Self-control | Model 1 | -0.205 | 0.019 | 0.000 | -0.243 | -0.167 | 0.041 |
|  | Model 2 | -0.217 | 0.022 | 0.000 | -0.260 | -0.173 | 0.056 |
|  | Model 3 | -0.073 | 0.032 | 0.024 | -0.135 | -0.010 | 0.005 |
| Sexual risk taking | Model 1 | 0.103 | 0.022 | 0.000 | 0.061 | 0.146 | 0.011 |
|  | Model 2 | 0.060 | 0.025 | 0.018 | 0.010 | 0.109 | 0.022 |
|  | Model 3 | 0.002 | 0.037 | 0.965 | -0.071 | 0.074 | 0 |
| Aggression | Model 1 | 0.166 | 0.020 | 0.000 | 0.127 | 0.205 | 0.028 |
|  | Model 2 | 0.126 | 0.022 | 0.000 | 0.082 | 0.170 | 0.063 |
|  | Model 3 | 0.043 | 0.031 | 0.171 | -0.019 | 0.105 | 0.002 |
| Alcohol use | Model 1 | 0.052 | 0.023 | 0.020 | 0.008 | 0.097 | 0.003 |
|  | Model 2 | 0.048 | 0.026 | 0.067 | -0.003 | 0.100 | 0.028 |
|  | Model 3 | -0.033 | 0.035 | 0.337 | -0.102 | 0.035 | 0.001 |
| Cannabis use | Model 1 | 0.020 | 0.033 | 0.549 | -0.045 | 0.085 | 0 |
|  | Model 2 | 0.007 | 0.037 | 0.859 | -0.066 | 0.079 | 0.022 |
|  | Model 3 | -0.053 | 0.062 | 0.390 | -0.175 | 0.068 | 0.003 |
| Benefit status | Model 1 | 0.061 | 0.022 | 0.005 | 0.018 | 0.105 | 0.004 |
|  | Model 2 | 0.038 | 0.026 | 0.141 | -0.012 | 0.088 | 0.028 |
|  | Model 3 | -0.034 | 0.030 | 0.259 | -0.093 | 0.025 | 0.001 |
| Anxiety | Model 1 | 0.132 | 0.022 | 0.000 | 0.089 | 0.176 | 0.018 |
|  | Model 2 | 0.113 | 0.025 | 0.000 | 0.064 | 0.163 | 0.046 |
|  | Model 3 | 0.056 | 0.030 | 0.067 | -0.004 | 0.116 | 0.003 |
| Conflict with law | Model 1 | 0.074 | 0.024 | 0.002 | 0.027 | 0.121 | 0.005 |
|  | Model 2 | 0.044 | 0.026 | 0.091 | -0.007 | 0.096 | 0.032 |
|  | Model 3 | -0.017 | 0.037 | 0.635 | -0.090 | 0.055 | 0 |
| Anti-social behaviour | Model 1 | 0.085 | 0.021 | 0.000 | 0.043 | 0.127 | 0.007 |
|  | Model 2 | 0.073 | 0.025 | 0.003 | 0.024 | 0.122 | 0.026 |
|  | Model 3 | -0.054 | 0.037 | 0.144 | -0.125 | 0.018 | 0.003 |
| Employment status | Model 1 | -0.008 | 0.020 | 0.701 | -0.046 | 0.031 | 0 |
|  | Model 2 | -0.009 | 0.022 | 0.696 | -0.052 | 0.034 | 0.003 |
|  | Model 3 | 0.037 | 0.029 | 0.193 | -0.019 | 0.093 | 0.001 |
| Income | Model 1 | -0.082 | 0.026 | 0.002 | -0.133 | -0.031 | 0.007 |
|  | Model 2 | -0.061 | 0.029 | 0.034 | -0.117 | -0.005 | 0.036 |
|  | Model 3 | -0.01 | 0.042 | 0.804 | -0.093 | 0.072 | 0 |

# Table S4. Prediction of adulthood developmental outcomes at age 23 years from child-reported, subjective household chaos at age 12 years in regression models

|  | Model | β | S.E. | p | CI 95% | | R2 |
| --- | --- | --- | --- | --- | --- | --- | --- |
| Education | Model 1 | -0.198 | 0.017 | 0.000 | -0.231 | -0.165 | 0.039 |
|  | Model 2 | -0.125 | 0.018 | 0.000 | -0.161 | -0.089 | 0.190 |
|  | Model 3 | -0.005 | 0.025 | 0.840 | -0.053 | 0.043 | 0.000 |
| Depression | Model 1 | 0.132 | 0.016 | 0.000 | 0.100 | 0.164 | 0.017 |
|  | Model 2 | 0.108 | 0.019 | 0.000 | 0.071 | 0.145 | 0.045 |
|  | Model 3 | 0.054 | 0.023 | 0.018 | 0.009 | 0.099 | 0.003 |
| Self-control | Model 1 | -0.161 | 0.016 | 0.000 | -0.192 | -0.131 | 0.026 |
|  | Model 2 | -0.141 | 0.018 | 0.000 | -0.177 | -0.105 | 0.038 |
|  | Model 3 | -0.065 | 0.023 | 0.005 | -0.111 | -0.020 | 0.004 |
| Sexual risk taking | Model 1 | 0.075 | 0.018 | 0.000 | 0.039 | 0.111 | 0.006 |
|  | Model 2 | 0.041 | 0.021 | 0.056 | -0.001 | 0.082 | 0.016 |
|  | Model 3 | 0.017 | 0.030 | 0.574 | -0.042 | 0.075 | 0.000 |
| Agression | Model 1 | 0.132 | 0.017 | 0.000 | 0.099 | 0.164 | 0.017 |
|  | Model 2 | 0.072 | 0.019 | 0.000 | 0.035 | 0.109 | 0.053 |
|  | Model 3 | 0.026 | 0.023 | 0.252 | -0.019 | 0.071 | 0.001 |
| Alcohol use | Model 1 | 0.042 | 0.018 | 0.017 | 0.008 | 0.077 | 0.002 |
|  | Model 2 | 0.040 | 0.021 | 0.057 | -0.001 | 0.082 | 0.024 |
|  | Model 3 | 0.020 | 0.027 | 0.456 | -0.033 | 0.073 | 0.000 |
| Cannabis use | Model 1 | 0.056 | 0.023 | 0.016 | 0.010 | 0.103 | 0.003 |
|  | Model 2 | 0.045 | 0.026 | 0.087 | -0.007 | 0.096 | 0.023 |
|  | Model 3 | 0.014 | 0.038 | 0.713 | -0.060 | 0.088 | 0.000 |
| Benefit status | Model 1 | 0.051 | 0.017 | 0.003 | 0.017 | 0.084 | 0.003 |
|  | Model 2 | 0.015 | 0.019 | 0.426 | -0.022 | 0.053 | 0.029 |
|  | Model 3 | 0.006 | 0.022 | 0.801 | -0.038 | 0.049 | 0.000 |
| Anxiety | Model 1 | 0.076 | 0.018 | 0.000 | 0.042 | 0.111 | 0.006 |
|  | Model 2 | 0.075 | 0.020 | 0.000 | 0.036 | 0.113 | 0.035 |
|  | Model 3 | 0.044 | 0.022 | 0.051 | 0.000 | 0.087 | 0.002 |
| Conflict with law | Model 1 | 0.059 | 0.017 | 0.000 | 0.026 | 0.091 | 0.003 |
|  | Model 2 | 0.017 | 0.021 | 0.406 | -0.024 | 0.059 | 0.030 |
|  | Model 3 | 0.038 | 0.026 | 0.146 | -0.013 | 0.089 | 0.001 |
| Anti-social behaviour | Model 1 | 0.080 | 0.016 | 0.000 | 0.048 | 0.112 | 0.006 |
|  | Model 2 | 0.054 | 0.020 | 0.008 | 0.014 | 0.093 | 0.024 |
|  | Model 3 | 0.044 | 0.024 | 0.062 | -0.002 | 0.090 | 0.002 |
| Employment status | Model 1 | -0.006 | 0.016 | 0.685 | -0.037 | 0.024 | 0.000 |
|  | Model 2 | -0.014 | 0.017 | 0.403 | -0.047 | 0.019 | 0.003 |
|  | Model 3 | -0.026 | 0.023 | 0.245 | -0.071 | 0.018 | 0.001 |
| Income | Model 1 | -0.037 | 0.022 | 0.095 | -0.081 | 0.006 | 0.001 |
|  | Model 2 | -0.030 | 0.025 | 0.242 | -0.079 | 0.020 | 0.034 |
|  | Model 3 | -0.024 | 0.039 | 0.541 | -0.099 | 0.052 | 0.001 |

# Table S5. Prediction of adulthood developmental outcomes at age 23 years from child-reported, subjective household chaos at age 9 years in regression models

|  | Model | β | S.E. | p | CI 95% | | R2 |
| --- | --- | --- | --- | --- | --- | --- | --- |
| Education | Model 1 | -0.170 | 0.023 | 0.000 | -0.215 | -0.124 | 0.029 |
|  | Model 2 | -0.040 | 0.025 | 0.121 | -0.089 | 0.010 | 0.195 |
|  | Model 3 | -0.026 | 0.032 | 0.421 | -0.089 | 0.037 | 0.001 |
| Depression | Model 1 | 0.084 | 0.022 | 0.000 | 0.042 | 0.126 | 0.007 |
|  | Model 2 | 0.044 | 0.025 | 0.075 | -0.004 | 0.093 | 0.037 |
|  | Model 3 | 0.019 | 0.030 | 0.528 | -0.040 | 0.078 | 0.000 |
| Self-control | Model 1 | -0.111 | 0.022 | 0.000 | -0.155 | -0.068 | 0.012 |
|  | Model 2 | -0.081 | 0.026 | 0.002 | -0.132 | -0.030 | 0.024 |
|  | Model 3 | -0.029 | 0.032 | 0.368 | -0.092 | 0.034 | 0.001 |
| Sexual risk taking | Model 1 | 0.053 | 0.025 | 0.035 | 0.004 | 0.103 | 0.003 |
|  | Model 2 | 0.039 | 0.028 | 0.170 | -0.017 | 0.094 | 0.013 |
|  | Model 3 | 0.008 | 0.041 | 0.842 | -0.071 | 0.088 | 0.000 |
| Aggression | Model 1 | 0.117 | 0.022 | 0.000 | 0.074 | 0.161 | 0.014 |
|  | Model 2 | 0.052 | 0.025 | 0.037 | 0.003 | 0.101 | 0.051 |
|  | Model 3 | 0.018 | 0.031 | 0.553 | -0.042 | 0.078 | 0.000 |
| Alcohol use | Model 1 | 0.049 | 0.025 | 0.047 | 0.001 | 0.097 | 0.002 |
|  | Model 2 | 0.019 | 0.028 | 0.495 | -0.036 | 0.074 | 0.028 |
|  | Model 3 | -0.006 | 0.037 | 0.864 | -0.079 | 0.066 | 0.000 |
| Cannabis use | Model 1 | 0.063 | 0.034 | 0.065 | -0.004 | 0.130 | 0.004 |
|  | Model 2 | 0.032 | 0.038 | 0.391 | -0.042 | 0.106 | 0.025 |
|  | Model 3 | -0.085 | 0.054 | 0.120 | -0.191 | 0.022 | 0.007 |
| Benefit status | Model 1 | 0.063 | 0.023 | 0.007 | 0.017 | 0.108 | 0.004 |
|  | Model 2 | 0.025 | 0.026 | 0.337 | -0.026 | 0.076 | 0.030 |
|  | Model 3 | 0.029 | 0.031 | 0.342 | -0.031 | 0.089 | 0.001 |
| Anxiety | Model 1 | 0.084 | 0.023 | 0.000 | 0.038 | 0.130 | 0.007 |
|  | Model 2 | 0.072 | 0.027 | 0.008 | 0.019 | 0.124 | 0.036 |
|  | Model 3 | 0.024 | 0.032 | 0.462 | -0.040 | 0.087 | 0.001 |
| Conflict with law | Model 1 | 0.049 | 0.021 | 0.022 | 0.007 | 0.090 | 0.002 |
|  | Model 2 | 0.019 | 0.023 | 0.407 | -0.064 | 0.026 | 0.033 |
|  | Model 3 | 0.020 | 0.030 | 0.502 | -0.038 | 0.078 | 0.000 |
| Anti-social behaviour | Model 1 | 0.078 | 0.024 | 0.001 | 0.030 | 0.125 | 0.006 |
|  | Model 2 | 0.032 | 0.030 | 0.285 | -0.027 | 0.090 | 0.026 |
|  | Model 3 | 0.031 | 0.035 | 0.369 | -0.037 | 0.099 | 0.001 |
| Employment status | Model 1 | -0.022 | 0.022 | 0.314 | -0.064 | 0.021 | 0.000 |
|  | Model 2 | -0.007 | 0.027 | 0.780 | -0.060 | 0.045 | 0.003 |
|  | Model 3 | -0.017 | 0.036 | 0.630 | -0.087 | 0.053 | 0.000 |
| Income | Model 1 | -0.043 | 0.029 | 0.142 | -0.101 | 0.014 | 0.002 |
|  | Model 2 | -0.025 | 0.035 | 0.472 | -0.093 | 0.043 | 0.033 |
|  | Model 3 | 0.057 | 0.044 | 0.194 | -0.029 | 0.144 | 0.003 |

# Table S6. Prediction of adult developmental outcomes from children’s subjective household chaos from age 9 through 16 years in twin difference models

|  | β | S.E. | p | CI 95% | | R2 |
| --- | --- | --- | --- | --- | --- | --- |
| Education | 0.013 | 0.025 | 0.610 | -0.036 | 0.061 | 0.000 |
| Depression | 0.058 | 0.023 | 0.010 | 0.014 | 0.102 | 0.003 |
| Self-control | -0.088 | 0.023 | 0.000 | -0.133 | -0.042 | 0.008 |
| Sexual risk-taking | 0.032 | 0.029 | 0.277 | -0.026 | 0.089 | 0.001 |
| Aggression | 0.053 | 0.023 | 0.023 | 0.007 | 0.099 | 0.003 |
| Alcohol use | 0.024 | 0.027 | 0.365 | -0.028 | 0.077 | 0.001 |
| Cannabis use | 0.000 | 0.040 | 0.996 | -0.079 | 0.078 | 0.000 |
| Benefits | -0.007 | 0.023 | 0.772 | -0.051 | 0.038 | 0.000 |
| Anxiety | 0.055 | 0.024 | 0.020 | 0.009 | 0.101 | 0.003 |
| Conflict with law | 0.017 | 0.024 | 0.490 | -0.031 | 0.065 | 0.000 |
| Antisocial behaviour | 0.044 | 0.024 | 0.063 | -0.002 | 0.091 | 0.002 |
| Employment status | 0.006 | 0.024 | 0.798 | -0.042 | 0.054 | 0.000 |
| Income | 0.000 | 0.044 | 0.996 | -0.086 | 0.086 | 0.000 |

# Table S7. Prediction of adult developmental outcomes from parent-reported household chaos at children’s ages 9 through 14 years in twin difference models

|  | β | S.E. | p | CI 95% | | R2 |
| --- | --- | --- | --- | --- | --- | --- |
| Education | -0.239 | 0.017 | .000 | -0.272 | -0.205 | 0.057 |
| Depression | 0.135 | 0.017 | .000 | 0.102 | 0.169 | 0.018 |
| Self-control | -0.111 | 0.016 | .000 | -0.143 | -0.080 | 0.012 |
| Sexual risk-taking | 0.098 | 0.019 | .000 | 0.061 | 0.135 | 0.010 |
| Aggression | 0.143 | 0.017 | .000 | 0.110 | 0.175 | 0.020 |
| Alcohol use | 0.028 | 0.018 | .128 | -0.008 | 0.064 | 0.001 |
| Cannabis use | 0.042 | 0.025 | .092 | -0.007 | 0.092 | 0.002 |
| Benefits | 0.095 | 0.017 | .000 | 0.063 | 0.128 | 0.009 |
| Anxiety | 0.067 | 0.017 | .000 | 0.033 | 0.101 | 0.005 |
| Conflict with law | 0.101 | 0.018 | .000 | 0.066 | 0.136 | 0.010 |
| Antisocial behaviour | 0.074 | 0.016 | .000 | 0.042 | 0.105 | 0.005 |
| Employment status | -0.002 | 0.016 | .901 | -0.033 | 0.029 | 0.000 |
| Income | -0.063 | 0.023 | .006 | -0.108 | -0.018 | 0.004 |

# Additional analyses

We compared our twin-difference models in the overall sample to those in samples of monozygotic and dizygotic twins, respectively. The regression estimates differed negligibly across zygosity. Note that these models clustering regression error terms at the family level to account for the correlation of error terms within families is not possible when fitting models to subsamples of monozygotic and dizygotic twins only. For three adulthood outcomes – educational attainment, depression, conflict with the law – we observed significant regressions in one but not the other zygosity group (Table S8). In particular, the prediction of educational attainment and depression in adulthood was significant in dizygotic twins but not in monozygotic twins and the overall sample. The prediction of conflict with the law was significant in monozygotic twins but not in dizygotic twins and the overall sample. To test if these differences are meaningful, rather than due to spurious effects, we also conducted multi-group models that restricted the regression paths from twins’ differences in the perception of household chaos at age 16 to their differences in adulthood outcomes to be equal across zygosity.

## Table S8. Twin difference model predictions of twins’ differences in subjective household chaos at age 16 for adult outcomes in monozygotic versus dizygotic twins

|  | **Dizygotic twins** | | | | | | **Monozygotic twins** | | | | | |
| --- | --- | --- | --- | --- | --- | --- | --- | --- | --- | --- | --- | --- |
|  | β | S.E. | p | CI 95% | | R2 | β | S.E. | p | CI 95% | | R2 |
| Education | 0.126 | 0.054 | 0.021 | 0.019 | 0.232 | 0.016 | 0.033 | 0.041 | 0.425 | -0.113 | 0.048 | 0.001 |
| Depression | 0.161 | 0.048 | 0.001 | 0.067 | 0.256 | 0.026 | 0.059 | 0.042 | 0.163 | -0.024 | 0.141 | 0.003 |
| Self-control | -0.145 | 0.049 | 0.003 | -0.242 | -0.049 | 0.021 | -0.135 | 0.043 | 0.002 | -0.219 | -0.051 | 0.018 |
| Sexual risk-taking | -0.024 | 0.061 | 0.689 | -0.143 | 0.095 | 0.001 | 0.059 | 0.049 | 0.224 | -0.036 | 0.154 | 0.003 |
| Aggression | 0.183 | 0.048 | 0.000 | 0.090 | 0.277 | 0.034 | 0.102 | 0.041 | 0.012 | 0.023 | 0.183 | 0.034 |
| Alcohol use | 0.111 | 0.055 | 0.045 | 0.003 | 0.219 | 0.012 | 0.147 | 0.048 | 0.002 | 0.053 | 0.241 | 0.022 |
| Cannabis use | 0.187 | 0.115 | 0.105 | -0.039 | 0.412 | 0.035 | 0.055 | 0.076 | 0.474 | -0.095 | 0.204 | 0.003 |
| Benefits | -0.027 | 0.052 | 0.599 | -0.129 | 0.075 | 0.001 | -0.033 | 0.041 | 0.426 | -0.048 | 0.114 | 0.001 |
| Anxiety | 0.143 | 0.055 | 0.009 | 0.035 | 0.251 | 0.020 | 0.109 | 0.045 | 0.015 | 0.021 | 0.196 | 0.012 |
| Conflict with law | -0.011 | 0.054 | 0.845 | -0.117 | 0.096 | 0.000 | 0.126 | 0.046 | 0.006 | 0.036 | 0.216 | 0.016 |
| Antisocial behaviour | 0.155 | 0.063 | 0.013 | 0.032 | 0.278 | 0.024 | 0.108 | 0.048 | 0.024 | 0.014 | 0.202 | 0.012 |
| Employment status | -0.053 | 0.051 | 0.302 | -0.153 | 0.048 | 0.000 | -0.032 | 0.042 | 0.450 | -0.114 | 0.051 | 0.001 |
| Income | -0.018 | 0.076 | 0.808 | -0.167 | 0.130 | 0.000 | -0.040 | 0.066 | 0.544 | -0.170 | 0.089 | 0.002 |

The results of the multi-group models suggested no meaningful differences in the results between monozygotic and dizygotic, because all model fit comparison statistics were not significant (Table S9).

## Table S9: Multi-group model fit comparisons across monozygotic and dizygotic twins

|  | **Chi-square diff** | **p-value** |
| --- | --- | --- |
| Education | 3.8317 | 0.05029 |
| Depression | 2.3614 | 0.12440 |
| Conflict with the law | 3.0925 | 0.07866 |

Note. Chi-square diff refers to the model fit difference between the configural invariance model (i.e., no restrictions across groups) and models where regressions were restricted to be equal between monozygotic and dizygotic twins.
